# The complete chloroplast genome of a medical herb, *Potentilla parvifolia* Fisch. (Rosaceae), from Qinghai-Tibet Plateau in China

**DOI:** 10.1080/23802359.2020.1866447

**Published:** 2021-02-08

**Authors:** Jinping Li, Likuan Liu, Hongyu Wang, Caiming Li, Wenming Zuo, Yang Zeng

**Affiliations:** a The College of Biological Science, Qinghai Normal University, Xining, China; b Academy of Plateau Science and Sustainability, Xining, China

**Keywords:** *Potentilla parvifolia*, chloroplast genome, Rosaceae, Qinghai-Tibet Plateau, phylogenetic trees

## Abstract

*Potentilla parvifolia* Fisch. (Rosaceae) is one of the genuine medicinal materials in Qinghai-Tibet Plateau, China. Here we report the first chloroplast (cp) genome of *P. parvifolia* using Illumina NovaSeq 6000 platform. The length of its complete cp genome is 152,898 bp, containing four sub-regions; a large single copy region (LSC) of 84,160 bp and a small single copy region (SSC) of 18,128 bp are separated by a pair of inverted repeat regions (IRs) of 25,305bp. The complete cp genome of *P. parvifolia* contains 130 genes, including 85 protein-coding genes, 37 tRNA genes, and 8 rRNA genes. The overall GC content of the cp genome is 37.2%. The phylogenetic analysis, based on 17 cp genomes, suggested that *P. parvifolia* is closely related to *P. fruticosa* L. and *Fragaria* species.

*Potentilla parvifolia* belongs to the family Potentilla of Rosaceae, it is a typical deciduous shrub in China alpine areas (Northwest Institute of Plateau Biology, Chinese Academy of Sciences 1987). *Potentilla parvifolia*, is one of the common Tibetan herbal medicines used in the treatment of indigestion and lung disease. It is mainly distributed in meadows and forest margins area (altitude 2500–4200 m) in Qinghai, Tibet, Neimenggu, Gansu Provinces and other places in China (Yao et al. 2011).

To study the systematic position and genetic background of *P. parvifolia*, we sequenced the *P. parvifolia* DNA and obtained its complete chloroplast (cp) genome. The voucher specimen of *P. parvifolia* was collected from the meadow in the Xianmi forest farm in Menyuan County, Qinghai Province, China, on 11 August 2019 (alt. 2762 m, E102°1′12.37″, N37°14′44.91″), and the specimen deposited at Herbarium, School of Life Sciences, Zhengzhou University, the voucher number is ZZU2019-6306, and it stored in Qinghai-Tibetan Plateau Museum of Biology. The total DNA was isolated from leaf materials of the voucher specimen using the plant genomic DNA extraction kit (Solarbio Life Sciences, China). The DNA concentration and quality were then measured by NanoDrop2000c micro-UV spectrophotometer (Thermo Scientific, America). The DNA was sequenced at Novogene Biotech Co. (Beijing, China) using the Illumina NovaSeq 6000 platform with a 150-bp shotgun library. In the end, 1.98 G of 150-bp paired-end raw reads of *P. parvifolia* were obtained, processed and assembled following the method of Nicolas et al. (2017). The assembled contigs were mapped to the reference cp genome (*Dasiphora fruticosa*, GenBank accession no. NC_036423) (The name has been amended to *P. fruticosa* L.) and annotated using Geneious Prime software (https://www.geneious.com) (Kearse et al, 2012). The border regions between the large single copy region (LSC), the small single copy region (SSC) and two inverted repeat regions (IRs) were validated by PCR amplifications and Sanger sequencing. The complete cp genome of *P. parvifolia* is 152,898 bp in length (GenBank accession no. MT942676). It contains two IRs of 25,305 bp, separated by a LSC of 84,160 bp and a small SSC of 18,128 bp. The cp genome of *P. parvifolia* is comprised of 130 genes, including 85 protein-coding genes, 8 rRNA genes, and 37 tRNA genes. The overall GC content of the cp genome is 37.2%, while the corresponding values of the LSC, SSC, and IR regions are 35.2%, 31.1%, and 42.9%.

The cp genome of *P. parvifolia* and 16 cp genome sequences (downloaded from GenBank) were aligned using MAFFT (Katoh and Standley 2013) and constructed phylogenetic trees using maximum likelihood (model: Tamura-Nei model) and neighbor-joining (model: Maximum Composite Likelihood) methods in MEGA7 (Kumar et al. 2016). *Malus toringoides* (Rosaceae) and *Eriobotrya japonica* (Rosaceae) were selected as outgroups. The results showed that *P. parvifolia* was sister group to *P. fruticosa* and they were close related to Fragaria species (Figure 1). The phylogenetic analysis was consistent with previous studies (Dobes and Paule 2010).

**Figure 1.**
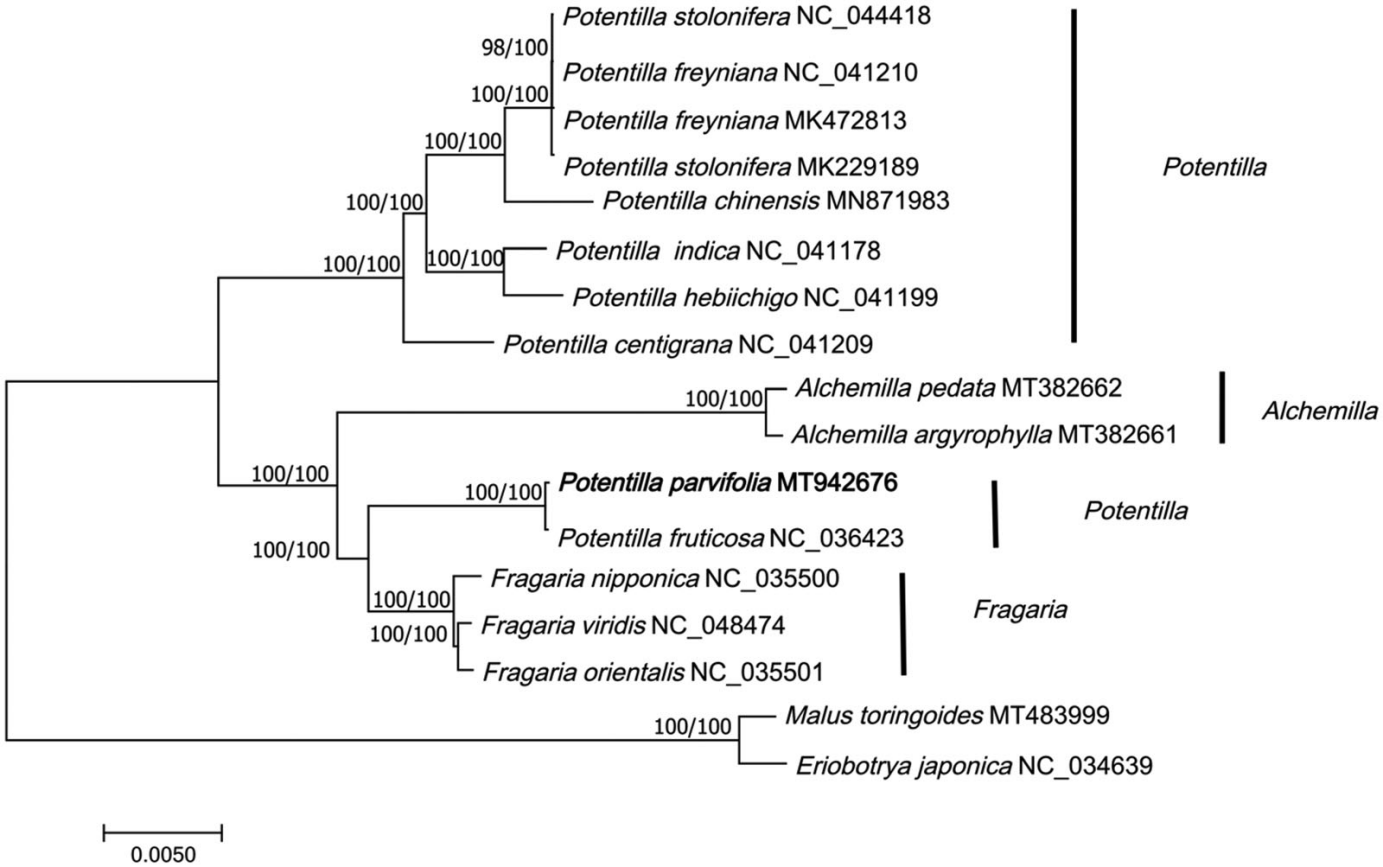
Phylogenetic tree of 17 species based on complete chloroplast genome sequences using NJ (with 1000 replicates) and ML (with 1000 replicates) methods. The numbers below the branches indicate the corresponding bootstrap support values from the ML and NJ trees. *Malus toringoides* (MT483999) and *Eriobotrya japonica* (NC_034639) are outgroups.

## Data Availability

The data that support the findings of this study are openly available in NCBI at https://www.ncbi.nlm.nih.gov/, reference number [MT942676], or available from the corresponding author.
